# Practical Notes on Diagnosis and Treatment

**Published:** 1906-12-08

**Authors:** 


### Practical Notes on Diagnosis and Treatment.

**Rheumatism and Pneumonia.**

In the course of severe attacks of rheumatic fever in young adults it is not very rare to get pneumonia as one incident of the illness.—*Sir Thos. Barlow.*

**Pruritus Vulvæ.**

The following is recommended as a local application: Potassium bromide and lupulin, of each 2 parts; calomel, 10 parts; olive oil, 30 parts. Apply occasionally after well shaking the bottle.

**Boracite in Renal Calculus.**

In cases of renal colic not admitting of removal of the stone by operation, boracite in the form of pulv. magnesii et boro-citratis is useful. A teaspoonful in a half-pint of hot water should be taken on rising, and again in the afternoon. I have in my possession many specimens of eroded calculi and of *débris* passed by patients when on this treatment. However the drug acts, it certainly helps to remove concretions and gravel from the upper part of the urinary tract.—*Mr. Reginald Harrison.*

**Pain in Cancer of the Breast.**

While it is an undeniable fact that cancer of the breast in its latest stages is almost always a terribly painful disease, the early stages are almost always invariably painless. In the majority of cases a period of twelve months to two years elapses between the commencement of the disease and the appearance of pain sufficient to cause much complaint.—*Sir Mitchell Banks.*

**Treatment of Ear Discharges.**

In an uncomplicated case the meatus should be cleansed with a solution of perchloride of mercury (1 in 2,000), and then, after drying, a few drops of the following may be introduced: Boric acid, 10 grains; glycerine, 1 drachm; rectified spirit, 1 oz. If this fails, or if the meatus is sodden and irritable, the dry treatment may be tried. This consists of blowing into the meatus (after cleansing and drying) finely powdered boric acid or iodoform, either alone or mixed with alum.

